# A Systematic Review on Nerve-Related Adverse Effects following Mandibular Nerve Block Anesthesia

**DOI:** 10.3390/ijerph19031627

**Published:** 2022-01-31

**Authors:** Luca Aquilanti, Marco Mascitti, Lucrezia Togni, Maria Contaldo, Giorgio Rappelli, Andrea Santarelli

**Affiliations:** 1 Department of Clinical Specialistic and Dental Sciences, Università Politecnica delle Marche, Via Tronto 10/A, 60126 Ancona, Italy; l.aquilanti@pm.univpm.it (L.A.); togni.lucrezia@gmail.com (L.T.); g.rappelli@staff.univpm.it (G.R.); andrea.santarelli@staff.univpm.it (A.S.); 2 Multidisciplinary Department of Medical-Surgical and Dental Specialties, University of Campania “Luigi Vanvitelli”, Via Armanni, 5, 80138 Naples, Italy; maria.contaldo@gmail.com; 3 Dentistry Clinic, National Institute of Health and Science of Aging, IRCCS INRCA, Via Tronto 10/A, 60126 Ancona, Italy

**Keywords:** inferior alveolar nerve block, anesthesia, nerve injury, paresthesia, prolonged anesthesia, adverse effects

## Abstract

Inferior alveolar nerve (IAN) block injections are commonly used in clinical practice, but they are not free from complications. The aim of the present systematic review is to assess the nerve-related adverse effects of IAN block anesthesia. A structured and systematic search was performed on the major electronic databases (PubMed, Cochrane Library, Web of Science, Scopus and CINAHL) for studies published in English until 30 September 2021. A total of 131 articles were identified through database searching using combinations of keywords. Fifteen papers were included and assessed for eligibility. Overall, nerve damage following an IAN block anesthesia injection is a rare occurrence, probably due to the direct nerve trauma of the needle, a neurotoxic effect of the used anesthetic solution and/or a combination of them. From a medico-legal point of view, a balanced discussion prior to nerve block anesthesia should be pursued in order to avoid patients’ reluctance to undergo necessary dental treatment due to the remote eventuality of nerve injury.

## 1. Introduction

The temporary decrease in the perception of pain during dental treatments is able to reduce the onset of anxiety among dental patients, and it is fundamental in clinical practice [1]. Local anesthetics are reliable and efficient drugs, but clinicians should be aware that complications may occur [2].

When performing a local anesthetic technique in a dental setting, systemic to loco-regional complications may arise. According to Haas [3], adverse events due to anxiety are the most common phenomena associated with local anesthetic injection. The affected subject usually experiences syncope, but a wide range of symptoms may appear as well (e.g., hyperventilation, nausea, vomiting and heart rate or blood pressure alteration). Additionally, allergic reactions may happen, even if the incidence of allergies to amide local anesthetics is less than 1% [4]. These manifestations should be also put in differential diagnosis with anxiety-induced events [5]. In addition, a local anesthetic may be toxic if a high concentration of the agent is reached in the bloodstream, especially if multiple injections are performed or because of an inadvertent intravascular injection [6]. Another adverse reaction, mainly associated with some anesthetic agents (e.g., prilocaine, articaine or the topical anesthetic benzocaine), is methemoglobinemia. This event is caused by an excess of anesthetic agent metabolites, resulting in systemic cyanosis [7]. Prolonged anesthesia or paresthesia of the tongue or lip has been documented as well. It is mostly transient, but it may become permanent with the lingual nerve more commonly affected than the inferior alveolar nerve. Other reported complications comprise: (i) facial nerve paralysis due to the inoculation of an anesthetic agent into the capsule of the parotid gland, with a transient inability to close the ipsilateral eye, with an intact corneal reflex; (ii) post-injection trismus, possibly due to hematoma formation, infection, multiple injections or an excessive volume of local anesthetics; (iii) pain from the inoculation of the local anesthetic agent, caused by the rapid injection of the anesthetic into the tissue or by the shape of the needle; (iv) post-injection infection, due to the contamination of the needle; (v) needle fracture and other complications such as edema, hematoma, gingival lesions, soft tissue injury and taste alteration [2,3].

Overall, injection methods and anesthetic solutions are the most important factors when performing local anesthesia, as they play a decisive role in the success of anesthesia itself [8]. The Halstead, Vazirani–Akinosi and Gow-Gates techniques are some of the described techniques available for the anesthesia of the inferior alveolar nerve (IAN) [9]. An IAN block is commonly used in dental practice, but a high failure rate has been reported, reaching 20–47% [10,11]. Even if these techniques reported different success and failure rates, they are still used on the basis of the clinical situation and practitioners’ comfortability [12]. Moreover, many expediencies were proposed to enhance IAN block success rates, such as developing new anesthetics, changing the patient’s position, and adjusting the drug dosage [11,13,14,15]. Regarding anesthetic solutions, articaine was reported as being more likely than others to be associated with paresthesia. Nevertheless, it is considered a safe local anesthetic for clinical use in dentistry since it can be used safely and effectively in both adults and children [16].

Currently, it is not possible to provide an accurate estimate of the incidence of adverse phenomena due to IAN anesthetic techniques. Thus, the aim of the present systematic review is to assess the nerve-related adverse effects of IAN block anesthetic injection techniques.

## 2. Materials and Methods

This systematic review was performed in accordance with the recommendations of the “Preferred Reporting Items for Systematic Reviews and Meta-Analyses Protocols (PRISMA-P) statement” [17]. In accordance with the guidelines, the present systematic review protocol was registered in the International Prospective Register of Systematic Reviews (PROSPERO) (registration number CRD42022282622).

### 2.1. Information Sources

A structured and systematic search was performed on the major electronic databases for studies published in the English language until 30 September 2021: PubMed, Cochrane Library, Web of Science, Scopus and CINAHL databases. Handsearching of the reference lists of included studies, relevant reviews, national clinical practice guidelines or other relevant documents was performed. Moreover, the Database of Abstracts of Reviews of Effects (DARE) was searched to examine any further existing systematic reviews and meta-analyses assessing the adverse effects related to IAN block injections.

### 2.2. Search Strategy

The following keywords were used in order to perform database searches: “mandibular”, “nerve injuries”, “nerve block”, “adverse effects”, “prolonged anesthesia”, “paresthesia” and “dysesthesia”, in combination with the Boolean operators “AND” and “OR”. A pilot search was undertaken in order to ensure that the search strategy was effective. The study focused on the Population/Patient, Intervention, Control/Comparison, Outcome(s) (P.I.C.O.S.) criteria [18]. In particular, studies involving humans who experienced IAN block anesthesia in either a private or public dental office were included in the present review. Subjects of any age, sex, ethnicity, socio-economic status and comorbidities were considered. Studies reporting IAN block anesthesia-related adverse effects were included. The adverse effects were defined as undesirable outcomes that occur during or after the use of a drug or intervention but are not necessarily caused by it [19]. Any type of adverse event affecting either the mandibular or lingual nerve, or both, was examined. The primary outcome of the review was the assessment of the related adverse effects, in terms of alteration in nerve sensation, in the IAN block and their estimated incidence. The secondary outcomes were: (i) the identification of the most usual types of anesthetic drugs related to undesirable effects, as well as the concentration of their anesthetic agent; (ii) the evaluation of the most affected nerve; (iii) the hypothesized explanation of the mechanism that leads to nerve damage.

### 2.3. Eligibility Criteria

Eligible studies were (a) studies published in the English language; (b) studies published in a peer-reviewed journal; (c) studies published until 30 September 2021; (d) clinical studies; I studies reporting non-surgical nerve injuries. Studies were excluded if they were: (a) reviews, editorials, commentaries, letters, book chapters, reports on prospective ideas and futuristic scenarios (protocols included) and dissertations. 

### 2.4. Data Extraction 

All the eligible citations were imported into a bibliographic software or citation management system and duplicates were removed. Selected papers were then imported into Rayyan QCRI (Qatar Computing Research Institute (Data Analytics), Doha, Qatar) for screening. Two reviewers (L.A. and M.M.) carried out the evaluations independently. The very first selection was made on the basis of papers’ title or abstract, and eligible ones were selected for full-text review. Titles and abstracts were screened by two independent review authors for assessment against the inclusion criteria for the review. Full-text studies that did not meet the inclusion criteria were excluded, and the reasons for exclusion were provided in the systematic review. At each stage of the study selection process, if a consensus was not reached, a third review author (A.S.) was consulted. The results of the search were reported in the final report and presented in a PRISMA flow diagram.

For the assessment of each publication, Excel spreadsheets were compiled. Data were extracted using a standardized form which included (a) authors’ names and the year of publication, (b) the country in which the study was performed, (c) the study design, (d) the aim of the studI, (e) the sample size, (f) the mean age ± standard deviation or age range, (g) the used anesthetic solution, (h) the retrieved article main outcomes and (i) the retrieved article secondary outcomes. Both authors compared their assessments and confirmed the data on the basis of the compiled spreadsheets. In case of doubt, concerning the study data, the two reviewers resolved disagreements by discussion. In the case of doubt, a third reviewer solved discrepancies. 

### 2.5. Quality Assessment

The methodological quality of the studies included was assessed with the Cochrane Collaboration tool for assessing the risk of bias, as suggested in the Cochrane Handbook for Systematic Reviews of Interventions (version 6.2). The currently recommended risk-of-bias tools, such as the RoB 2 tool for randomized trials and the ROBINS-I tool for non-randomized studies, were used [20,21]. The critical appraisal checklist for case reports provided by the Joanna Briggs Institute (JBI) was used to perform a quality assessment of the studies [22]. 

## 3. Results

**Figure 1:**
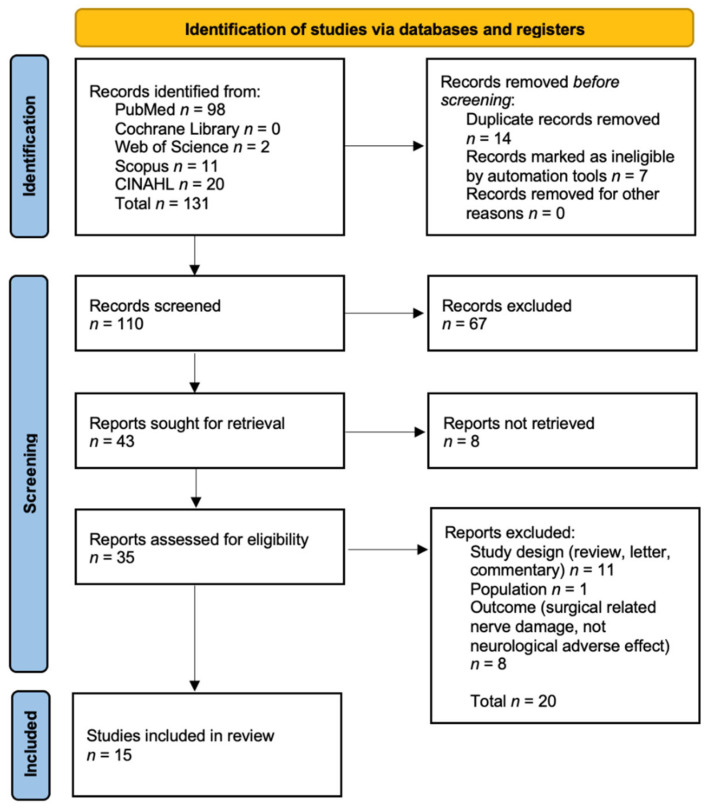
The flowchart of the search results and selected studies was constructed on the basis of the Preferred Reporting Items for Systematic Reviews and Meta-Analyses (PRISMA) flowchart [23].

A total of 131 articles were identified through database searching using combinations of keywords (PubMed *n* = 98, Cochrane Library *n* = 0, Web of Science *n* = 2, Scopus *n* = 11, CINAHL *n* = 20). Out of 131 papers, 14 were excluded as they were duplicates, and 7 were discarded because they were written in a language different from English (French *n* = 4, Russian *n* = 2 and Dutch *n* = 1). A total of 110 articles were further reviewed, assessing the coherency of titles and abstracts with the aim of the present review. During this stage, 67 records were excluded, due to several reasons: wrong population (e.g., studies on cadavers and on animal models), wrong study design (e.g., letters, commentaries and systematic reviews), wrong outcome (e.g., needle breakage). After abstract reviews, 43 articles were selected for further inspection. Out of 43 papers, 8 were not retrieved and, for this reason, excluded from the review. Thirty-five articles were assessed for eligibility. A total of 20 papers were excluded because they did not meet the inclusion criteria (wrong publication type *n* = 11; wrong outcome *n* = 8; wrong population *n* = 1). A total of 15 articles were included in the present systematic review (Figure 1). 

The studies included in the present review were conducted in nine different countries: the USA (*n* = 4), Australia (*n* = 2), Canada (*n* = 2), Denmark (*n* = 2), France (*n* = 1), Germany (*n* = 1), India (*n* = 1), Italy (*n* = 1) and Japan (*n* = 1). Full-text articles that met the eligibility criteria are included in Table 1. Overall, nine retrospective studies, as well as two prospective studies, three case reports and one case series, were included in the present systematic review.

### 3.1. Prospective Studies

According to Krafft and Hickel, 18 patients out of 12,104 (0.15%) showed a lingual sensory disturbance, with a complete recovery after 6 months in 94.5% of the cases. Moreover, 1.04% of the patients included in that study complained of other disturbances such as trismus (0.4%), lasting pain in the area of treatment (0.3%) or swelling of the cheek (0.08%). In 0.08% of the cases, lingual sensory disturbances and pain arose at the same time [24]. An estimated incidence of permanent nerve involvement of 1:160,571 inferior alveolar nerve blocks was reported in the study by Pogrel and Tamby [25]. In this study, the LN was affected in 79% of patients and the IAN in 21% of patients. Moreover, 56.6% of the patients experienced a very painful injection or felt an electric shock sensation during the inferior alveolar nerve block injection, while 30.1% reported no discomfort during the injection. When considering the reported symptoms, most of the patients (66.3%) complained of paresthesia or anesthesia as the most annoying symptom, while others (30.1%) reported dysesthesia as the predominant symptom. 

Overall, the subjects included in both the studies received one or a combination of the following anesthetic solutions: articaine with or without epinephrin, lidocaine with or without epinephrin, lidocaine with octapressin, butanilicaine, mepivacaine with or without epinephrin, prilocaine and bupivacaine [24,25]. Prilocaine was found to be more frequently linked to cases of nerve involvement in the study by Pogrel and Tamby [25], while there was no relation between the type of anesthetic itself and the lingual sensory deficit in the study by Krafft and Hickel [24].

### 3.2. Retrospective Studies

Overall, the included studies evaluated cases of nerve damage caused by IAN block injection techniques during a 24-year-long period (1987–2011) in either children or adults. Different anesthetic solutions with different concentrations were considered with articaine, prilocaine, lidocaine, mepivacaine, bupivacaine or a combination of them being the most used ones. Four studies reported the incidence of nerve involvement related to the anesthetic injection, being 1:609,000 in the study by Gaffen and Haas [29], 1:13,800,970 in that by Garisto et al. [30], 1:750,000 in that by Pogrel et al. [27] and 1:27,415 in that by Sambrook and Goss [32]. The lingual nerve (LN) appeared to be the most damaged nerve, followed by the IAN and chorda tympani (CTN). The most common complaints related to IAN block injection techniques were: (i) paresthesia of the tongue, chin, cheek and lower lip; (ii) dysesthesia; (iii) taste disturbance (especially caused by CTN damage); (iv) allodynia; (v) prolonged anesthesia; (vi) burning sensation; (vii) hyperesthesia; (viii) pain or electric shock sensation during the injection procedure. Paresthesia and dysesthesia ranged from mild to severe, and no cases of total anesthesia were recorded [33]. Overall, either complete or partial nerve recovery may happen within more than 2 years. However, a permanent paresthesia may occur, especially if no signs of recovery appear after more than 9 months [33]. 

When considering the anesthetic type and its concentration, articaine and prilocaine were the two drugs mostly associated with a higher frequency of paresthesia. In particular, Garisto et al. reported that 4% anesthetic solutions (prilocaine and articaine) were more highly associated with the development of paresthesia than those of a lower concentration [30]. Moreover, anesthetic solutions with 4% articaine caused 54% of the observed cases of sensory impairment in the study by Hillerup and Jensen [28]. Similarly, significative overrepresentation of NSDs was associated with 4% articaine. A study reported that prilocaine was associated with 34% of cases of nerve damage, followed by articaine and lidocaine, with 33% and 25%, respectively [33]. However, the risk of permanent nerve damage related to inferior alveolar nerve block may occur with any local anesthetic, with varying incidences [33]. Piccinni et al. also reported a significant disproportionality of paresthesia, oral paresthesia and dysesthesias when using articaine and prilocaine [34]. Finally, Sambrook and Goss reported that all cases of prolonged anesthesia were related to lignocaine [32].

### 3.3. Case Series and Case Report

Overall, cases involving five males and four females were described. Bendgude et al. reported a case of prolonged anesthesia in a 4-year-old male patient: due to the prolonged anesthesia, a ulcerative lesion of the lower lip as well as a scratch was assessed, with satisfactory healing after 2 weeks [38]. Chevalier et al. described a case of Bell’s palsy in a 34-year-old female patient. Two hours after the inferior alveolar nerve block, the patient experienced a complete and fast paralysis of the left-sided facial muscles, with a subtotal recovery and a persistent slight muscular stiffness 1 year after the injection [37]. Hotta et al. presented two cases involving two females (41 and 22 years old) in which taste disturbance occurred, associated with atrophy of the fungiform papillae and either a burning sensation or a numbness sensation. The prognosis was favorable for recovery within 1 year from taste disturbance due to inferior alveolar nerve damage during administration of the dental anesthetic [36]. Finally, Kingon et al. described adverse effects that occurred in a sample of five subjects (four males and one female, mean age: 56.8 ± 8.7) who complained of dysesthesia, a severe dysesthetic sensation, a mild paresthetic sensation and taste alteration associated with other symptoms (e.g., electric shock sensation at the site of the injection, painful numbness, difficulty in talking and eating), following inferior alveolar nerve block injection. The authors argued that there is an increased risk of prolonged anesthesia of the IAN and, mostly, of the LN when higher concentrations of local anesthetic agents are used [35].

### 3.4. Quality Assessment

The quality of the studies included in the review was evaluated using the protocols described by Sterne et al. [21] and by the JBI [22]. The available literature to include in the present review article was restricted as the studies were judged to have a serious risk of bias in at least one bias domain, but not a critical bias in any domain. The case reports and the case series retrieved for this review were included in the analysis as they were evaluated as worthy to be included in the study. 

## 4. Discussion

As previously stated, the IAN block anesthesia technique is a safe and common procedure used all over the world, but it is not free from complications. One of them is represented by the possible damage that may affect nerves, mostly the LN, the IAN and the CTN. Nerve damage may determine the types of alteration and degrees of sensitivity of the respective areas of innervation. Overall, trigeminal nerve lesions and their clinical consequences represent an important challenge in modern dentistry, especially in oral surgery. The relevance of the symptomatology that afflicts the patient in the case of damage to a sensitive nerve and the inevitable consequences for the professional determine the main issue of this type of lesion, especially from a medico-legal point of view.

Unfortunately, an accurate estimate of the exact incidence of the phenomenon is not possible due to the underestimation of such eventualities and the lack of reliable data on the number of anesthesia procedures performed. Moreover, the fact that some articles were older than 10 years may have limited the results of the present systematic review. However, the limitation was not imposed on the time span of publication because the authors deemed the included papers relevant for the purpose of the present study. Generally, reviews that only rely on published data may lead to poor and inconsistent outcome definitions and assessments, due to the limited information available from this type of report on incompletion or lack of specificity. Golder et al. investigated published versus unpublished studies, finding that a median of 43% of published studies reported adverse event data, in comparison to a median of 83% of unpublished studies [39]. Furthermore, the studies were retrospective, prospective, case reports and case series. Therefore, the data are difficult to quantify, as most of these injuries may be not reported. A possible explanation of this may lay in the fact that the professional tends to wait for the patient to accept the damage, in the hope that this will fade over time. Therefore, the results of the present systematic review should be interpreted with caution. Overall, few studies reported the incidence of nerve-related adverse effects as a result of an IAN block injection, ranging from 1:27,415 in the study by Sambrook and Goss [32] to 1:13,800,970 in that by Garisto et al. [30]. Other studies reported an estimated incidence of nerve involvement of 1 in 609,000 and 1:750,000 IAN blocks [27,29]. Both men and women may almost equally experience anesthesia-related adverse effects, with a slight predominance of female patients with post-injection paresthesia. Interestingly, even though no statistical difference was found in the injured mandibular side, there was a slight tendency for post-injection adverse effects on the side opposite to the clinician’s used hand due to a greater difficulty in delivering the injection [26]. Anyway, these estimates may be underestimated as nerve damage could be not reported. 

Other important aspects to consider are the type and concentration of anesthetic solution used, as these may influence the risk of nerve damage. In particular, articaine and prilocaine are considered the two drugs most commonly associated with a higher frequency of nerve disturbance, especially when 4% solutions are used. Garisto et al. indicated that 4% anesthetic solutions are more commonly associated with the development of paresthesia than those of a lower concentration [30]. Similarly, Hillerup and Jensen reported that anesthetic solutions with 4% articaine caused more than half of the observed cases of sensory impairment [28]. It was speculated that 4% formulations may cause a double nerve injury: one due to the neurotoxicity of the anesthetic agent, and the other caused by the direct trauma caused by the needle [31]. Piccinni et al. confirmed the higher rate of paresthesia, oral paresthesia and dysesthesias when using articaine and prilocaine [34]. However, a recent systematic review, aimed at comparing the efficacy and the safety of different local anesthetic agents for mandibular third molar extraction, reported that the most effective local anesthetic is 4% articaine, compared with 2% lidocaine, 0.5% bupivacaine and 1% ropivacaine. The same authors reported that lidocaine is the safest local anesthetic, although all investigated solutions can be used safely [14]. 

Overall, the clear mechanism of nerve injury is still debated [25]. Two different theories have been proposed, namely: the direct trauma of the injection needle to either the nerve or the intraneural blood vessels, and the neurotoxicity of the local anesthetic [40]. The first one implies the direct contact of the injection needle with the nerve, traumatizing it and determining a prolonged alteration in sensation. Once the tip of the needle contacts the bone, it may become barbed, enhancing the risk for perineurium rupture, endoneurium herniation and the transection of multiple nerve fibers and entire fascicles, especially on withdrawal [41,42]. However, the injection needle may also traumatize the intraneural blood vessels, determining an intraneural hematoma. The hemorrhage may lead to constrictive epineuritis, compressing the nerve fibers within the rigid tissue confines and causing localized neurotoxicity [27,41]. In addition, hematoma formation may determine the onset of reactive fibrosis and the formation of a scar, inhibiting the natural healing of the nerve [26,43]. Finally, the second theory hypothesizes that the anesthetic solution itself may cause chemical damage to the nerve, especially if it is placed intrafascicularly, causing demyelination, axonal degeneration and inflammation of the surrounding nerve fibers within the fascicles [25,44,45]. 

Most of the subjects who experienced nerve-related adverse effects following an IAN block reported an electric shock-like or burning sensation [24,25,26,27,28,29,30,32,34,35,36,37]. The explanation for this type of symptomatology could be the direct contact of the injection needle with part of the nerve, but it is not indicative of permanent nerve injury [27]. In fact, while the occurrence of this type of sensation has been estimated between 1.3% and 8% of all IAN block injections, only a little more than half of the subjects suffering from a prolonged altered sensation experienced this symptom during the injection [40]. The LN appears to be the most involved nerve when considering nerve damage (the LN was affected in 70% of cases versus 30% of cases for the IAN). Pogrel et al. speculated that the LN and IAN differ in their fascicular pattern above the lingula, where an IAN block is performed [46]. Overall, nerves are made of parallel bundles of nerve fibers, called fascicles. The nerve fascicles vary in size, number and pattern among nerves and along their anatomical path. In particular, the LN may be unifascicular, being at risk of being injured more easily and permanently than a multifascicular nerve, which may have a greater recovery power [46]. Overall, nerve damage determines the onset of a range of symptoms, whose severity ranges from mild to severe, in a transient or permanent way. If a diagnosis of paresthesia is made, the clinician should examine the patient for the distribution of the sensory loss/disturbance and monitor continually. Generally, local anesthetic injection-induced IAN or LN dysfunction spontaneously reverts in 85–94% of patients [25,47]. However, if no recovery or improvement occurs within two weeks, referral to a specialist with experience in nerve injuries is recommended [32,43]. If a subject experienced paresthesia for more than 8 weeks after the initial injury, full recovery is less probable and a permanent paresthesia may occur, if no signs of recovery appear after more than 9 months [27,33,48].

## 5. Conclusions

The results of the present systematic review suggest that nerve damage following an IAN block anesthesia injection is a rare occurrence. Even if a clear mechanism of nerve injury has not been established yet, all clinicians should be aware that nerve damage may occur as a result of the direct nerve trauma of the needle, a neurotoxic effect of the used anesthetic solution and/or a combination of them. From a legal point of view, the risk of nerve damage is not usually discussed during consent prior to nerve block anesthesia, especially not for surgical treatments. The patients’ information on possible nerve damage can be moderate as its occurrence is very unlikely to happen. In addition, if the patient does not receive balanced information on the risks/benefits of the procedures, the patient may decide to avoid the dental treatment due to the remote eventuality of nerve injury. However, if it occurs, it must be documented early, and clinicians should avoid denying responsibility or promise that it will resolve soon. Referral to a specialist is recommended.

## Figures and Tables

**Table 1:** Summary of the studies included in the review (list of abbreviations: LN = lingual nerve; IAN = inferior alveolar nerve; ADR = adverse drug reaction; NSD = neurosensory disturbance; EGM = electrogustometry; FPD = filter paper disk; F = female; M = male).

Author (Year)	Country	StudyDesign	Aim	Sample Size	Age (Years)	Drug	Main Outcome	Secondary Outcome
Krafft and Hickel (1994) [24]	Germany	Prospective Study (1987–1990)	To investigate the amount of damage to the LN by mandibular block anesthesia alone.	12,104 block anesthetics (5637 in F and 6467 in M)	-	Articaine (39.7%) Articaine + adrenalin (0.2%) Lidocaine + adrenalin (57.3%) Lidocaine + octapressin (1.3%) Butanilicaine (1.0%) Lidocaine + adrenalin (0.1%) Mepivacaine (0.1%) Mepivacaine + adrenalin (0.1%) Missing data (0.2%)	Immediate electric shock sensation upon needle insertion in 876 patients. Rate of 0.15% of lingual sensory disturbance.	Other complaints following nerve block were trismus (*n* = 49), pain (*n* = 39), swelling (*n* = 10) and other not specified complaints (*n* = 28). No relation between the type of anesthetic itself and the lingual sensory deficit.
Pogrel and Tamby (2000) [25]	USA	Prospective Study	To report what appeared to be permanent nerve involvement after receiving an IAN block	83 (55 F and 28 M)	41.2 (range: 21–83)	Lidocaine (*n* = 33). Prilocaine (*n* = 32). Mepivacaine (*n* = 3). Prilocaine + lidocaine (*n* = 6). Prilocaine + mepivacaine (*n* = 2). Prilocaine + etidocaine (*n* = 1). Mepivacaine + lidocaine (*n* = 1). Mepivacaine (×3) + bupivacaine (×2) + lidocaine (*n* = 1).	47 patients either reported a painful injection or felt the electric shock sensation. 28 patients reported dysesthesia as their most troublesome symptom, while 55 patients reported paresthesia or anesthesia as predominant. Estimated incidence of permanent nerve involvement of 1:160,571 IAN blocks. Prilocaine was more frequently linked to nerve involvement.	5 patients underwent surgery, but no obvious damage to the nerve was seen. The pain was worse after the surgery (*n* = 2). The symptoms of dysesthesia were a deep, boring, burning pain and occasional flushing over the associated cheek (*n* = 5). The dysesthesia appeared to have spread to involve other trigeminal nerve trunks (*n* = 6). Residual nerve involvement from mild to complete anesthesia (*n* = 2).
Harn and Durham (1990) [26]	USA	Retrospective Study (Clinical Survey)	To investigate the incidence of LN trauma and its associated complications when the conventional mandibular block technique is used.	2289 adults (1245 F, 1044 M)	32.1 (F) and 31.9 (M)	A total of 9587 mandibular block injections were performed.	A total of 206 patients reported 347 traumatic episodes one or more times in the past 5 years. The incidence of LN trauma after a mandibular block injection was 3.6% (347/9587). 9.0% of patients have had a traumatic episode to their lingual nerve during conventional mandibular block anesthesia. There is a 3.6% chance of traumatizing the LN every time a conventional mandibular block is given, with a 15.0% chance of a post-injection complication after a traumatic episode to the LN (paresthesia or prolonged anesthesia).	Of the patients who experienced traumatic episodes, 41 reported post-injection complications (19.9% of the patients who reported a traumatic episode and 1.8% of the patients within the study). Of the 347 mandibular blocks that traumatized the lingual nerve, 52 (15.0%) resulted in post-injection complications (0.5% of the mandibular blocks which led to post-injection complications). Duration of complication: ≤24 h (51.9%), 2–6 days (17.3%), 1–2 weeks (11.5%), 4 weeks (5.8%), 8 weeks (1.9%), 12 weeks (3.9%), 6 months (3.9%), >1 year (3.9%).
Pogrel et al. (1995) [27]	USA	Retrospective Study (1988–1992)	To report cases in which altered sensation occurred following injection of a local anesthetic.	12 (4 F and 8 M)	40 (range: 22–67)	2% lidocaine with 1:100,000 epinephrin (*n* = 8). 4% prilocaine with 1:200,000 epinephrine (*n* = 3). 2% mepivacaine with 1:20,000 levonordefrin (*n*= 1).	Nerve damage affected the LN in 9 cases and the IAN in 2 cases; in 1 most unusual case, only the chorda tympani was involved. Diagnosis of mild to severe nerve damage. Incidence of nerve involvement 1:75,000.	Electric shock-type sensation during injection (7 patients). The patient with chorda tympani damage reported not being able to taste anything over one half of the tongue. 1 patient reported complete recovery within 6 months and 3 patients within 12 months. The other 12 patients reported residual nerve damage after 18 months.
Hillerup and Jensen (2006) [28]	Denmark	Retrospective Study (1997–2004)	To clarify the magnitude of sensory impairment and the character of signs and symptoms in patients suffering sensory dysfunction after mandibular block analgesia. To follow and describe the level of function/dysfunction over time. To describe possible differences related to type of analgesic agent.	52 (35 F and 17 M)	47 (range: 24–81)	Articaine 4% Prilocaine 3% Lidocaine 2% Mepivacaine 3% Mepivacaine 3% + Articaine 4% An average volume of 2.6 mL analgesic solution was injected (range 1.4–12 mL).	The LN was more often injured, *n* = 42 (78%) than the IAN, *n* = 12 (23%). Neurogenic complaints of LN injury included paresthesia (*n* = 18), dysesthesia (*n* = 9), allodynia (*n* = 3), none (*n* = 3) and no other information/other (*n* = 9). IAN injury-related altered sensory function was reported as anesthesia (*n* = 2), hypesthesia (*n* = 6), hyperesthesia (*n* = 1) and normal sensory function + unpleasant neurogenic sensation (*n* = 3). Unpleasant (neurogenic) sensations included paresthesia (*n* = 8), dysesthesia (*n* = 2) and neuralgic pain (*n* = 1).	54% of the observed cases of sensory impairment were associated with articaine 4%. Most of the patients presented with both a neurosensory deficit and a neurosensory disturbance. Clinical signs of a neuroma (*n* = 9). Gustatory perception of the injured side was damaged (*n* = 33). No improvement in gustatory function over time (*n* = 18). Dysgeusia (*n* = 4) and 6 patients had trouble with the taste of salt (*n* = 6). 18 LN patients were re-examined, on average, 13 months after the injury: improved LN sensory function (*n* = 5), no difference (*n* = 2), deterioration of function (*n* = 2). No subjective data were obtained in 9 patients. Three IAN patients reported a painful electric shock on injection. Another 3 patients had no such experience, and in 6 patients, we have no data regarding sudden pain on injection. Four IAN patients were re-examined after an average of 8 months after the initial examination, showing no consistent change in IAN neurosensory function with time.
Gaffen and Haas (2009) [29]	Canada	Retrospective Study (1999–2008)	To analyze cases of paresthesia associated with local anesthetic injection.	182 (93 F and 89 M)	43.8 (range: 11–80)	Articaine (59.9%) Lidocaine (12.6%) Mepivacaine (3.3%) Prilocaine (15.9%) Multiple (8.2%)	The approximate incidence of non-surgical paresthesia in dentistry is 1:609,000. Injury solely to the LN occurred significantly more often than injury solely to the IAN.	Tongue paresthesia (79.7%) and lower lip/chin paresthesia (20.3%). Most affected areas by non-surgical paresthesia: tongue (79.1%); lower lip and chin area (28.0%); cheek (4.4%); tongue and lower lip/chin (9.9%). Altered taste sensation due to injury of chorda tympani nerve (14.3%). Painful/burning sensations (9.9%). Pain/electric shock sensation during injection (19.2%).
Garisto et al. (2010) [30]	Canada	Retrospective Study (1997–2008)	To determine if the type of local anesthetic administered had any effect on reports of paresthesia in dentistry in the United States.	248	41.9 (range: 15–78)	4% Articaine (51.3%) 4% Prilocaine (42.9%) 2% Lidocaine (4.9%) 0.5% Bupivacaine (0.4%) 3% Mepivacaine (0.4%)	248 cases of non-surgical paresthesia were reported (207 cases: mandibular nerve block, 2 cases: mental nerve block, 10 cases: infiltration). Incidence of 1:13,800,970. Paresthesia related to a local anesthetic injection alone is a rare event. The 4% prilocaine and articaine solutions are more associated with the development of paresthesia than those of lower concentration.	Articaine and prilocaine associated with a higher frequency of paresthesia. Male/female ratio = 0.63:1. Areas affected: tongue (*n* = 170), lower lip (*n* = 14), both (*n* = 7). Taste disturbance = 17.7% and dysesthesia = 21.8%. LN = 89.0%. Duration of paresthesia: from 1 to 736 days. Confirmed resolution of paresthesia: from less than 30 to 240 days.
Hillerup et al. (2011) [31]	Denmark	Retrospective Study (1995–2007)	To report ADRs and NSDs associated with injection of local anesthetics.	115 (81 F and 34 M)	47 (range: 23–80)	Articaine 4% Articaine and other anesthetics. Lidocaine 2% Mepivacaine 3% Prilocaine 3%	The NSDs affected a total of 131 branches of the trigeminal nerve (lingual *n* = 86, IAN *n* = 31, buccal *n* = 8, infraorbital *n* = 4, mental *n* = 2).	At the 1 follow-up examination, diagnosis of permanent NSDs (*n* = 85) and complete recovery (*n* = 5). Total of 25 patients (21.7%) did not attend their 1-year follow-up visit. Physical needle lesions are a major causative factor and indicate a causal link with properties of the injected substance. Overrepresentation of NSDs associated with 4% articaine related mainly to mandibular blocks. Overrepresentation of 4% articaine formulations in so-called “double injuries” indicates that properties of the injected substance are the causative agent through neurotoxicity.
Sambrook and Goss (2011) [32]	Australia	Retrospective Study (2009)	To review the literature regarding nerve injuries, to present recent data from South Australia and to discuss the management of local anesthetic-related nerve injuries.	8 (4 F and 4 M)	56.4 (range: 39–77)	Lidocaine. Lidocaine and epinephrine 1:80,000.	Incidence of prolonged anesthesia was 1: 27,415.	All cases of prolonged anesthesia related to lidocaine. Other complaints following nerve block were trismus (*n* = 3), pain (*n* = 4), taste alteration (*n* = 2), burning sensation (*n* = 2), numbness (*n* = 6). Six patients had resolution by 3 months, 2 patients had persistent altered sensation.
Pogrel (2012) [33]	USA	Retrospective Study (2006–2011)	To analyze cases of IAN and/or LN damage resulting from an IAN block.	41	-	Articaine Lidocaine Prilocaine Carbocaine	The symptoms included paresthesia and dysesthesias, varying from mild to severe, but there were no cases of total anesthesia.	Prilocaine is associated with 34% of cases, articaine with 33% and lidocaine with 25%. IAN blocks can cause permanent nerve damage with any local anesthetic, but the incidences may vary.
Piccinni et al. (2015) [34]	Italy	Retrospective Study (2004–2011)	To evaluate the possible alert signals of paresthesia by local anesthetics.	17,246 ADR	-	Lidocaine, bupivacaine, articaine, combinations of local anesthetics, prilocaine, ropivacaine, mepivacaine, cocaine, capsaicin, benzocaine, phenol, levobupivacaine, tetracaine, procaine, dyclonine, ethyl chloride, other local anesthetics	Paresthesia represented 46.9% of all local anesthetic-related reports, burning sensation 22.2%, oralparesthesia 13.9% and hyperesthesia 5.6%.	The highest number of reports was found for lidocaine (*n* = 247), followed by bupivacaine (*n* = 99), articaine (*n* = 85), combination of different local anesthetics (*n* = 45) and prilocaine (*n* = 30). More cases of paresthesia, oral paresthesia and dysesthesias were found with articaine and prilocaine.
Kingon et al. (2011) [35]	Australia	Case Series	To illustrate the impact of this prolonged anesthesia on patients’ quality of life.	5 (4 males and 1 females)	56.8 ± 8.7	Case 1, 2 and 4: 2.2 mL cartridge of 4% local anesthetic. Case 3: One cartridge of 4% local anesthetic for each side. Case 5: 4.4 mL of 3% local anesthetic (mepivacaine HCl).	Case 1: severe dysesthesia with some signs of recovery and taste alteration. Case 2: mild paresthesia which would resolve. Case 3: mild paresthesia which would resolve. Case 4: severe dysesthesia of the right lingual nerve with slow recovery and taste alteration. Case 5: severe lingual nerve injury with probable slow recovery and loss of taste.	Case 1: Electric shock sensation at the site of the injection. Dysesthesia continued but the mandibular nerve recovered. Difficulty in talking, eating and taste alteration. At 23 weeks after the injection, profound anesthesia of all of the right LN was found. There was burning sensation on contact and loss of taste. No improvement after 21 months. Case 2: Paresthesia of the right lingual nerve persisted, with the long buccal and mandibular nerves recovering sensation normally. Resolution of the paresthesia reported after 4 weeks. There was no interference with taste. There was still a mild paresthesia after 4 months. Case 3: The left side mandibular nerve recovered normally but on the right-side mental nerve, anesthesia persisted. Normal right and left LN sensation and no taste deficit. Improvement in paresthesia after 10 days. There was still a mild paresthesia after 4 months. Case 4: Electric shock sensation at the time of the injection. Dysesthesia persisted but the mandibular nerve recovered. Difficulty in talking, eating and had altered taste. The area of numbness slowly decreased. A 1 cm area on the right tongue tip was hypersensitive to contact. Taste was still altered. No further improvement after 2 years. Case 5: No pain at the injection site. The following day, the patient reported ongoing numbness and tingling of the right LN. The right mandibular nerve had fully resolved. No resolution after 1 year after the local anesthetic was administered.
Hotta et al. (2002) [36]	Japan	Case Report	To report two cases of temporary taste disturbance after inferior alveolar nerve block.	2 (F)	31.5 ± 13.4	-	Case 1: Taste disturbance and burning sensation on the left side of her tongue. Case 2: Numbness and taste disturbance on the right side of the tongue.	Case 1: No disturbance of tongue mobility and perception of pain. Touch was normal. The fungiform papillae on the left side were punctate and atrophied but those on the right side were normal. The only area with no response was that innervated by the left chorda tympani nerve. The results of both EGM and FPD test had normalized 11 months after the dental procedure. Case 2: Tongue moved normally but abnormal response on the right to temperature, pain and touch. No difference was noted in the fungiform papillae on the right and left sides. The patient’s temperature and pain sensation normalized 4 months after, but no improvement was detected in the results of EGM or FPD testing. At 15 months after dental procedure, the results of EGM and FPD testing were found to have returned almost to normal. Atrophy of fungiform papillae on the impaired side was detected after 5 ½ months. The prognosis is favorable for recovery within almost 1 year from taste disturbance due to IAN damage during administration of dental anesthetic. Taste disturbance ipsilateral to the side of anesthesia was presumed to have been caused by direct damage to the LN and chorda tympani nerves.
Chevalier et al. (2010) [37]	France	Case Report	To describe a case of complete unilateral Bell’s palsy.	1 (35 weeks pregnant F)	34	1.8 mL of chlorhydrate of mepivacaine	Diagnosis of Bell’s palsy.	Two hours after the IAN block, patient experienced paralysis of the left-sided facial muscles. After 1 year, subtotal recovery with persistent slight muscular stiffness was assessed.
Bendgude et al. (2011) [38]	India	Case Report	To report a complication of self-inflicted injury following IAN block.	1 (M)	4	-	Prolonged anesthesia.	Ulcerative lesion of the lower lip and scratch injury on the chin due to the numbness caused by the IAN block (healing after 2 weeks).

## Data Availability

The datasets generated and/or analyzed during the present study are available from the corresponding author on reasonable request.
